# Structure-Activity Relationships of Thiazolyl Resorcinols, Potent and Selective Inhibitors of Human Tyrosinase

**DOI:** 10.3390/ijms19030690

**Published:** 2018-02-28

**Authors:** Tobias Mann, Cathrin Scherner, Klaus-Heinrich Röhm, Ludger Kolbe

**Affiliations:** 1 Front End Innovation, Beiersdorf AG, 20245 Hamburg, Germany; tobias.mann@beiersdorf.com (T.M.); cathrin.scherner@beiersdorf.com (C.S.); 2 Institute of Physiological Chemistry, Philipps University, 35032 Marburg, Germany; roehm@staff.uni-marburg.de

**Keywords:** tyrosinase, human, inhibition, thiazolyl resorcinols, structure, activity

## Abstract

Tyrosinase inhibitors are of great clinical interest as agents for the treatment of hyperpigmentary disorders; however, most compounds described in the literature lack clinical efficiency due to insufficient inhibitory activity against human tyrosinase (hTyr). Recently, we reported that thiazolyl resorcinols (4-resorcinylthiazol-2-amines and -amides) are both selective and efficacious inhibitors of hTyr in vitro and in vivo. Here, we measured dose-activity profiles of a large number of thiazolyl resorcinols and analogous compounds to better understand the molecular basis of their interaction with hTyr. We show that both the resorcinyl moiety and the thiazole ring must be intact to allow efficient inhibition of hTyr, while the substituents at the thiazole 2-amino group confer additional inhibitory activity, depending on their size and polarity. The results of molecular docking simulations were in excellent agreement with the experimental data, affording a rationale for the structural importance of either ring. We further propose that a special type of interaction between the thiazole sulfur and a conserved asparagine residue is partially responsible for the superior inhibitory activity of thiazolyl resorcinols against hTyr.

## 1. Introduction

The tyrosinases (monophenol monooxygenases, EC 1.14.18.1) belong to the type-3 family of copper proteins, which also includes plant catechol oxidases and the O_2_-transporting hemocyanins of molluscs and arthropods. Tyrosinases catalyze the hydroxylation of monophenols to *o*-diphenols and the subsequent oxidation of the resulting *o*-diphenols to *o*-quinones using molecular oxygen as a co-substrate [1]. They are widely distributed in nature, where they have rather diverse functions. In animals, tyrosinase mediates the pigmentation of skin, eyes and hair by catalyzing the two-step oxidation of l-tyrosine to l-dopaquinone, which by further enzymatic and non-enzymatic steps eventually forms melanin [2]. Therefore, human tyrosinase is of considerable interest as the target of melanogenesis inhibitors which, in humans, have a potential to treat hyperpigmentation disorders such as melasma and solar lentigines [3]. Although hundreds of compounds of natural and synthetic origin have been proposed as tyrosinase inhibitors, it has turned out that most of them lack efficacy against human tyrosinase (hTyr), and thus only a few compounds are actually being in use for clinical and cosmetic purposes. The main reason for this situation is that so far most studies have only been using a commercially available tyrosinase from the mushroom *Agaricus bisporus* (mTyr, [4]), the substrate specificity of which is distinctly different from that of hTyr [5]. Although human tyrosinase can be isolated from melanomas [6,7,8], well-defined preparations of recombinant hTyr with activities sufficient for large-scale inhibition studies have become available only in recent years [9,10,11]. Moreover, in the last decade, several X-ray structures of tyrosinases and tyrosinase-like proteins have been published, including mTyr [12,13], bacterial tyrosinases from *Streptomyces castaneoglobisporus* (sTyr, [14]) and *Bacillus megaterium* (bTyr, [15]), respectively, and, most recently, the human tyrosinase-related protein 1 (hTrp1), a melanogenic protein of yet unknown function in humans [16]. Common structural features of these proteins have been reviewed by several authors [17,18,19]. However, the detailed three-dimensional structure of hTyr still remains to be elucidated.

In a recent study, we used a soluble hTyr construct expressed in human embryonic kidney (HEK-293) cells [9] to conduct a high-throughput screen (HTS) for hTyr inhibitors and found that thiazolyl resorcinols are potent and rather selective inhibitors of the human enzyme in vitro and of melanogenesis in vivo. We further showed that most compounds presently employed as melanogenesis inhibitors in vivo (including hydroquinone, kojic acid, and arbutin) are clearly inferior to thiazolyl resorcinols, at least in vitro [20]. In the present work, we carried out extensive inhibition studies to detect structure-activity relationships (SAR) in the thiazolyl resorcinol series. In addition, we employed virtual docking simulations of inhibitor binding to a homology model of hTyr to better understand the molecular interactions underlying the inhibition.

## 2. Results and Discussion

### 2.1. Structural Motifs Essential for Inhibition

**Figure 1:**
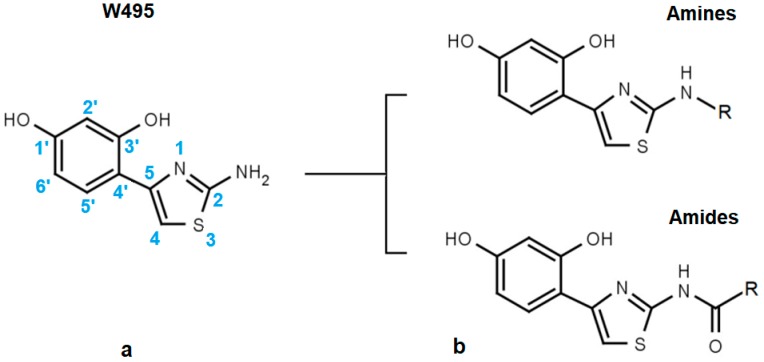
Basic structure of the resorcinyl thiazolamine inhibitors studied here is (**a**) the 4-(2-amino-1,3-thiazol-4-yl) resorcinol moiety. (**b**) Derivatization of the primary amino group of the resorcinyl thiazolamine W495 leads to either “Amines” or “Amides”, depending on the substituent.

The structural core of the inhibitors discussed here is a phenthiazamine derivative hydroxylated at the 1′ and 3′ positions of the phenyl ring (i.e., 4-(2-amino-1,3-thiazol-4-yl) resorcinol). This compound (Figure 1a) is a hTyr inhibitor with an inhibitor concentration at 50% inhibition (EC_50_) of about 50 µM. In our internal numbering system for tyrosinase inhibitors, it is called W495. Alkylation or acylation of the 2-amino group of the thiazole ring yields two further lines of active compounds that we refer to as “Amines” and “Amides” for brevity (Figure 1b). 

The resorcinol moiety is a well-known motif in tyrosinase inhibitors [21]. In fact, several resorcinol derivatives with alkyl-substituted 4′-carbon atoms are now being used for topical applications, e.g., 4-butylresorcinol [22,23,24], 4-hexylresorcinol [25], and 4-phenylethylresorcinol [26]. Here, we show that replacement of the 4′-alkyl substituent of these compounds with N-substituted 2-aminothiazole moieties can increase inhibitory potency against hTyr by a factor of 20 and more. Essential preconditions for an efficient inhibition of hTyr by thiazolyl resorcinols can be derived from Table 1, Table 2 and Table 3. The compounds shown are denoted by our internal code numbers (i.e., Wxxx), while inhibitory activity (as directed against the dopa oxidase activity of hTyr) is expressed as EC_50_, e.g., the half maximal effective inhibitor concentration calculated from dose-response curves. EC_50_ values above 3 mM cannot be reliably estimated by our assay; thus, a value of >3000 is given in these cases. All compounds shown here are competitive inhibitors of hTyr [20]. Therefore, their inhibition constants, K_i_, amount to about one third of the respective EC_50_ values.

Table 1a illustrates the effect of the 5-aryl moiety on inhibitory activity. Clearly, only a 1′,3′-dihydroxyphenyl (resorcinyl) substituent, as in compound W311, mediates efficient inhibition. With the exception of the phenolic compound W452, all other hydroxylation patterns of the phenyl ring fail to mediate inhibitory activity. The same holds when one of the ring OH-groups is replaced with fluorine such as in W480 

Similar conclusions regarding the importance of the thiazole ring can be drawn from Table 1b. Ring systems other than thiazole in that position impair inhibitory activity by a factor of at least 200. This holds for the isosteric imidazole ring (W633 vs. W547) as well as for benzene (W632 vs. W548), and triazole (W652, Table S1). While an inverted amide bond as in W607 only moderately increases EC_50_, the inverted thiazole ring in compound W701 abolishes activity.

While the structures of both rings are decisive for efficient inhibition, the nature of the substituents at the 2-amino group of the thiazole ring appears to be less critical. Many different kinds of groups are tolerated here, both in alkyl- or arylamines (Table 2a, left column) and in amides (Table 2a, right column). Generally, amides are more efficient than the corresponding amines by factors of 4–20. Moreover, there is a clear preference for small substituents in both series. Large and distinctly hydrophobic residues markedly impair inhibition (see, e.g., W496, W537, and W669 in Table 2b), while more hydrophilic substituents of the same size do not (compare, e.g., W537 with W570 or W669 with W680).

**Figure 2:**
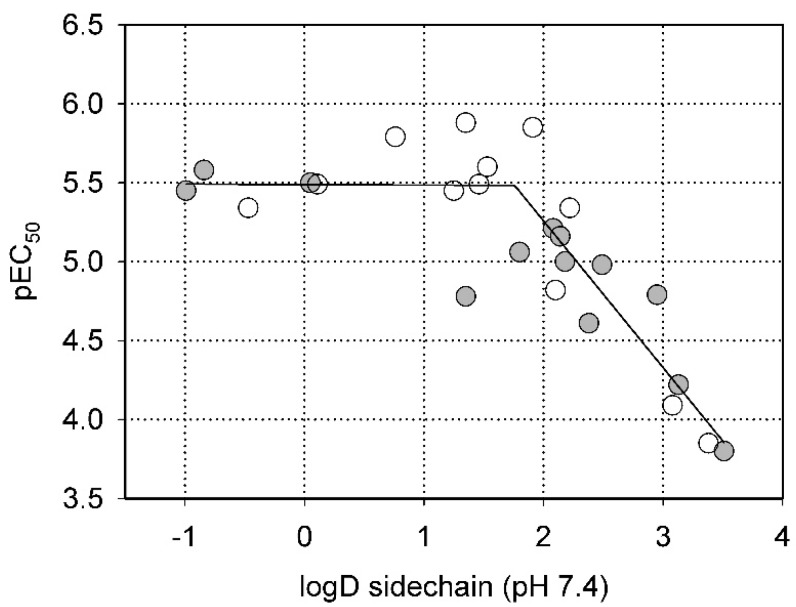
Dependence of the inhibitory activity of the compounds in Table 2 (as pEC_50_) on the polarity of the substituent at the 2-amino group of thiazole (expressed as partition coefficient, logD, at pH 7.4). Open circles—amides; filled circles—amines.

This conclusion is quantitatively illustrated by Figure 2, which shows the dependence of inhibitory activity on the polarity of the substituent at the 2-position of the thiazole ring. Clearly, in both series (amines and amides), more hydrophilic substituents with logD < 2 allow efficient inhibition, while pEC_50_ steadily decreases (i.e., EC_50_ increases) with logD values above that threshold.

A similar correlation between inhibitory activity and side chain polarity was seen with 4-alkyl resorcinols (Table 3a). Small hydrophobic substituents as in 4-butylresorcinol (W039) are preferred over longer or bulkier ones as in 4-hexylresorcinol (W072) or 4-phenylethylresorcinol (W038). Introduction of a terminal OH group into the 4-alkyl residue (W785) or methylation of the resorcinyl moiety between the hydroxy groups (W639), on the other hand, markedly impaired inhibition.

### 2.2. Steric Factors

In another series of experiments we compared thiazolyl resorcinols alkylated at various positions (Table 3b). Methylation of the thiazole 2-amino group only moderately reduced inhibitory activity in amines (compare W694 with W646) but strongly in amides (W548 vs. W605). Alkylation of either one of the aromatic rings (W548 vs. W625 and W681) abolished inhibition altogether. This suggests that strict steric requirements apply for effective binding of the thiazolyl resorcinol moiety.

### 2.3. Homology Modeling

**Figure 3:**
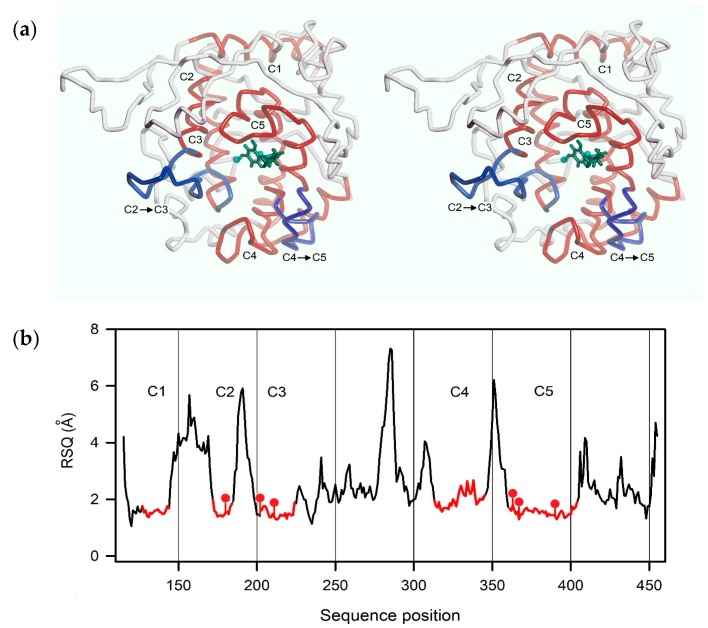
Structure of the homology model of hTyr used for docking simulations. (**a**) Backbone trace of the model (stereo representation). Core segments are highlighted in red, two relevant loops in blue (see text). The position of the active site is indicated by two copper ions (cyan balls) and a docked ligand (green); (**b**) Degree of structural conservation (expressed as residue-specific quality of an I-Tasser model (RSQ), see text) of each residue between model and templates. The highly conserved core segments C1 to C5 are shown in red.

Human tyrosinase is a monomeric glycoprotein anchored in the melanosomal membrane with the catalytic domain facing inward. The mature protein is made up of a compact globular domain (residues 18–456), a short segment (457–473) connecting the globular part to the transmembrane helix (predicted: 474–500), and a small cytosolic domain at the C-terminus (501–529). Recently, the architecture of the intramelanosomal domain of human tyrosinases became clear, when the crystal structure of the closely related tyrosinase-related protein 1 (Trp-1) was published [16,18]. As shown in Figure S1, the N-terminal cysteine-rich subdomain of the intramelanosomal domain (18–114), the function of which is still unknown, is tightly associated with the structurally conserved tyrosinase subdomain (115–456). A superposition of several tyrosinase structures (PDB entries 5m8p, 3npy, 1wx2, c2y9, 4j3p, and 4ou8) gave a root mean square deviation (RMSD) of only 2.0–2.3 Å for the tyrosinase subdomain. The central part of this domain harbors a characteristic core structure of five helices and three connecting loops that enclose the active site (colored red in Figure 3a). In this region, an even better structural alignment with a backbone RMSD of 0.8–1.1 Å was achieved. 

As three-dimensional structures of hTyr are still unavailable, we constructed homology models of the human enzyme to generate protein targets for molecular docking studies. To obtain initial models, we made use of web-based modeling servers, especially the I-Tasser suite [27]. I-Tasser ranks model quality according to several indicators, among them the so-called TM-score (Template Modeling Score), i.e., the weighted and normalized root mean square deviation (RMSD) between structurally aligned model and template. TM-scores above 0.5 indicate an identical overall fold. Another, residue-specific measure of alignment quality is the so-called residue-specific quality of an I-Tasser model (RSQ in Å), the estimated deviation of the residue in the model from the template.

As mentioned above, the construction of viable tyrosinase models is greatly facilitated by the presence of a highly conserved core that contains most of the active site. For this part of the models, I-Tasser and other modeling programs like Phyre2 [28] yielded excellent structural alignments. This is quantitatively illustrated by Figure 3b, which shows the RSQ (see above) for each residue of a top-ranking homology model of hTyr. The core segments that are denoted C1 to C5 include all six copper-coordinating histidines (red dots) and most of the residues interacting with thiazolyl resorcinol inhibitors (see Section 2.4).

The first homology models constructed for the present study were built before the X-ray structure of Trp-1 became available. They were mostly based on crystal structures of *B. megaterium* tyrosinase as the main templates (PDB entries 3nm8, 3npy, 4j6t and others), but also on tyrosinases from *A. oryzae* (3w6q), *A. bisporus* (mushroom, 2y9w), and catechol oxidase from *I. batatas* (1bt3). As the cysteine-rich part of hTyr is missing from bacterial and plant enzymes, the models only covered the tyrosinase subdomain. The top-ranking model had a high TM-score of 0.74 ± 0.11. When the Trp-1 structure was published, additional models were built. As expected, I-Tasser then selected Trp-1 structures (5m8l and others) as principal templates. While the optimum TM-score reached here was even higher (0.97 ± 0.15), the predicted structure of the core region were almost indistinguishable from that of the earlier model. Both the earlier and the later models were in good agreement with homology models published by other groups [18,29,30], especially with that of Favre et al. [30], for which molecular coordinates were published.

In order to construct a protein target suitable for molecular docking, the top-ranking hTyr model of the earlier series was reconstituted with a di-copper center in the conformation seen in *Bacillus* tyrosinase (PDB entry 3npy), and its amino acid side chains were energy-minimized. As seen in Figure 3a, loops connecting segment C2 with C3, and C4 with C5, respectively, flank the active site. As they show little sequence conservation within the tyrosinase family, and thus were not modeled consistently, these loops were subjected to molecular dynamic simulations to relax the structures locally and to explore possible conformations (for details, see Methods). A backbone trace of the final model is shown in Figure 3a. The carbohydrate chains of mammalian tyrosinases are essential for protein maturation, sorting and melanosomal targeting, but not required for enzymatic activity [31,32]. They were therefore omitted from the model.

### 2.4. Molecular Docking

Virtual docking simulations were carried out using Molegro Virtual Docker (MVD), an integrated platform for predicting protein-ligand interactions. MVD employs a fast and efficient docking strategy based on the highly accurate Moldock scoring function [33]. MVD’s docking solutions (“poses”) are rated by their Moldock scores (the sum of the intermolecular interaction energy between ligand and target, and the intramolecular energy of the ligand). In addition, a so-called rerank score (rSc) is calculated, which is a weighted combination of the MolDock score with additional steric terms. The more negative the rSc, the tighter the ligand binding. We therefore use the negative rerank score (nrSc) calculated by MVD as an indicator of predicted inhibitory activity.

**Figure 4:**
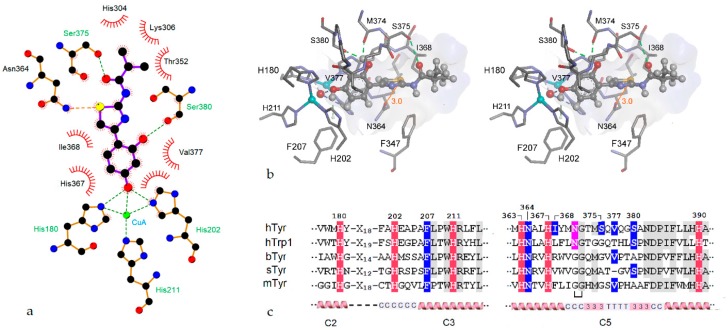
Virtual docking results for compound W630. (**a**) Interactions stabilizing the pose shown as LigPlot [34] diagram; (**b**) Stereo representation of the lowest-energy docking solution. Only amino acid residues directly interacting with the bound inhibitor are shown (residue numbering includes the signal peptide). The di-copper center with the bridging oxygen is visible on the left with copper ions depicted as cyan balls. The ligand is shown in ball-and-stick representation, amino acid residues as sticks. The inner surface of the binding cavity is colored according to hydrophobicity on a scale from blue for hydrophilic to brown for hydrophobic; (**c**) Partial alignments of the CuA (left) and CuB sites (right) of several tyrosinases or tyrosinase related proteins. Copper-chelating histidines are highlighted in red, residues in contact with the bound ligand in blue, and the glycosylation site N371 in pink. Amino acids conserved in at least 4 sequences are shaded gray. Secondary structure elements and core regions (see text) are shown on the bottom. hTyr—human tyrosinase; hTrp1—human tyrosinase-related protein; bTyr—*Bacillus megaterium* tyrosinase; sTyr—*Streptomyces castaneoglobisporus* tyrosinase; mTyr—mushroom (*Agaricus bisporus*) tyrosinase. The bracket below the mTyr sequences denotes an extra loop.

In-depth docking experiments were performed for about 30 of the compounds shown in Table 1, Table 2 and Table 3 (for details of the setup of the simulations, see Methods). It turned out that, for most members of the thiazolyl resorcinol series, rather similar docking solutions were obtained. As a typical example, Figure 4 illustrates the top-ranking pose of the efficient inhibitor W630 (Thiamidol, [20]) and its interactions with our hTyr model. Figure 4a depicts the main interactions between ligand and model as LigPlot scheme [34], while Figure 4b shows the relevant part of the complex in a stereo representation Figure 4c contains a multiple alignment of both copper-binding motifs of hTyr and a number of other tyrosinases for reference.

The aromatic ring of the resorcinol moiety is held in place by π–π interactions with histidine 367 and by hydrophobic interactions with Val377. This valine residue sits above the CuA site and is thought to act as so-called “blocker” residue, covering the active site and modifying the ratio of monophenolase and diphenolase activities [17]. The orientation of the resorcinol moiety seen in our model is almost the same as those of co-crystallized phenolic ligands in other tyrosinases and tyrosinase-like proteins such as tyrosine, l-Dopa, and tyrosinol in *Bacillus* tyrosinase (PDB entries 4p6r,4p6s,4p6t, cf. [15]) or tyrosine in Trp-1 (5m8p, [16]), indicating that the docking pose produced by our simulations is realistic. In addition to the noncovalent interactions that stabilize the resorcinol ring, hydrogen bonds connect its *p*-hydroxy group with the CuA center (i.e., with His202 and the bridging OH^−^ ion) while the *m*-hydroxy group forms hydrogen bonds with the side chain of Ser380 and the carbonyl oxygen of Met374 (cf. Figure 4b). Both residues are thought to play specific roles in catalytically active type 3 copper proteins. Ser380 might be essential for monophenol hydroxylation by binding the phenolic OH-group of the substrate [18], while the conserved Met374 is thought to stabilize the metal center and/or trap H_2_O_2_ molecules generated during tyrosinase catalysis [17].

With the exception of His202 and Phe207, the ligand-binding amino acids mentioned so far are all located within the highly conserved core structure C5. This segment is made up from two α-helices (residues 362–369, and 385–404, respectively) connected by a complex roof-like structure (residues 370–384), which covers the copper center and a part of docked ligands (cf. Figure 4b,c). The connecting structure contains two short 3_10_-helices that enclose a β-turn, and also harbors Asn371, one of the essential glycosylation sites of hTyr. It is well-conserved in both bacterial tyrosinases but interrupted by an extra loop in the mushroom enzyme.

At first glance, the interactions of the thiazole ring of the ligands with our enzyme model appear to be predominantly hydrophobic. The ring is located in a pocket that is confined on the distal side by the side chain of Ile368 and parts of the peptide backbone, and by the side chains of Asn346 and Phe347 below the ligand. As calculated by MVD, the interactions of the thiazole ring contribute about one third of the total interaction energy (cf. Table S1). However, as will be discussed in more detail below, this is probably an underestimation.

As mentioned above, small alkyl or acyl substituents with 3–4 carbons are preferred in the distal region of both amines and amides, while bulky, distinctly hydrophobic rings impair inhibitory activity. Our docking simulations provide a reasonable explanation for these observations. As shown by Figure 4b, substituents at the thiazole 2-amino group protrude beyond the active site cavity and thus are largely exposed to the aqueous solvent. Strongly hydrophobic moieties are therefore unfavorable due to the hydrophobic effect.

As already pointed out, the loops connecting core sections C2 with C3, and C4 with C5, respectively, are not well conserved, and therefore not modeled reliably (cf. Figure 3b). However, as suggested by our docking results, both loops do not make direct contacts with docked ligands and thus are probably not relevant for inhibitor binding.

### 2.5. Role of the Thiazole Ring

The distinguishing feature of the inhibitors considered here is the presence of a thiazole ring, which confers superior inhibitory activity to these compounds. As already mentioned, replacement of the thiazole moiety with other aromatic rings greatly impairs activity (cf. Table 1). This finding is not easily explained by chemical or steric properties of the rings in question and, indeed, MVD assigns similar binding energies to all of the rings examined (cf. Table S1).

**Figure 5:**
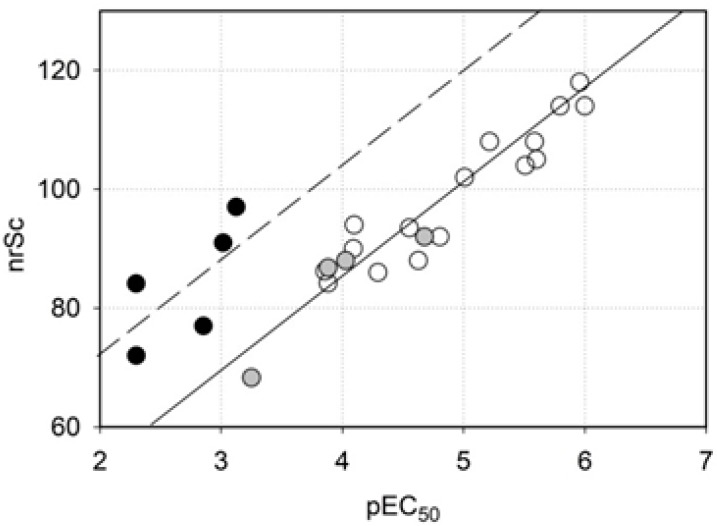
Inhibitory activity, as predicted by the negative rerank score (nrSc) provided by Molegro Virtual Docker (MVD), plotted against measured inhibitory activity (expressed as pEC_50_, the negative decadic logarithm of EC_50_). Open circles—amines and amides containing a thiazole ring; gray circles—4-alkyl resorcinols; black circles—compounds with other rings replacing the thiazole moiety.

**Figure 6:**
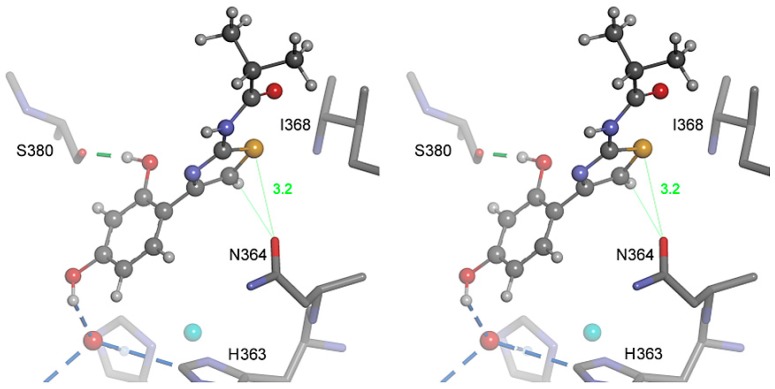
Proposed “sulfur bonding” interaction between the thiazole ring and Asn364 (stereo representation). The inhibitor W630 is shown in ball-and-stick representation, adjacent amino acid residues as sticks. The underlying structure is the same as shown in Figure 4a. For details, see text.

In Figure 5 the inhibitory activity predicted by MVD is plotted against measured activity. Clearly, there is an excellent correlation between these parameters in thiazolyl resorcinols of both series (open circles, *r*^2^ = 0.87), indicating that the homology model underlying the docking experiments is generally valid. A correlation of the same type is observed with 4-alkylresorcinols (gray circles). By contrast, compounds with rings different from thiazole (black circles) reached MolDock scores that were too high to fit into the scheme. On the other hand, the scores calculated for the thiazolyl resorcinols could just as well be too low. Recent publications about novel interactions between amino acids and sulfur-containing compounds [35,36] suggest that the latter interpretation may apply. Divalent sulfur atoms, especially in heterocyclic compounds such as thiazole, contain low-lying antibonding σ* orbitals that form a so-called “σ-hole”, i.e., a positive potential that can interact with electron-rich Lewis bases like oxygen or nitrogen atoms. This so-called “sulfur bonding” interaction, which has at least the strength of an H-bond, was shown to occur in many proteins [36]. In hTyr and other tyrosinases, the strictly conserved Asn364 is in an ideal position to participate in a sulfur bond with the thiazole sulfur. In our model, bonding angle and bonding distance (about 3.2 Å) are nearly optimal (Figure 6) for this type of interaction, indicating that it can significantly contribute to the strength and specificity of hTyr inhibition by thiazolyl resorcinols. As sulfur bonding is not implemented in the MVD force field, the calculated scores would indeed be too low for all thiazole-containing compounds.

## 3. Materials and Methods

### 3.1. Human Tyrosinase

A truncated, His-tagged form of hTyr (hTyr-D_His_) was expressed in HEK 293 cells and purified by metal affinity chromatography on Ni^2+^-Sepharose as described by Cordes et al. [9].

### 3.2. Sources of Inhibitors

4-Butylresorcinol was purchased from Vivimed Labs (Hyderabad, India). 4-Hexylresorcinol was obtained from Sigma-Aldrich (Taufkirchen, Germany). 4-Phenylethylresorcinol was provided by Witega Laboratorien (Berlin, Germany). All other resorcinol derivatives were either synthesized by Evotec Ltd. (Abingdon, UK), Witega Laboratorien (Berlin, Germany) or purchased from various suppliers via Scifinder (www.cas.org/products/scifinder). A complete documentation of the synthetic routes to and properties of all thiazolyl resorcinols is beyond the scope of this publication. However, by way of example, synthesis procedures for several alkylamidothiazoles are detailed in Supplementary Materials. Selected thiazolyl resorcinols are patented by Beiersdorf AG.

### 3.3. Tyrosinase Assay and Dose-Response Profiles

The l-Dopa oxidase activity of hTyr was assayed by a modification of the method of Winder and Harris [37]. In this assay, the reaction product, l-dopaquinone, is trapped by MBTH (3-methyl-2-benzothiazoline hydrazone) to yield a stable pink dye that can be quantified at 490 nm. Reaction mixtures in 100 mM phosphate buffer, pH 7.0, were set up in microtiter plates together with appropriate blanks to account for nonenzymatic oxidation of substrate and product. After thermal equilibration at 37 °C for 10 min, the reactions were started by adding enzyme and monitored for 10–20 min at 490 nm. Absorption values were read at 15 s intervals and digitally stored for later analysis. Dose-response curves were obtained by testing various concentrations of each inhibitor in triplicate at a fixed substrate (l-Dopa) concentration of 1 mM. Inhibitor concentrations at 50% inhibition (EC_50_ values) and their standard deviations were estimated by fitting a four-parameter logistic equation to the profiles using SigmaPlot 11 (Systat Software, San Jose, CA, USA).

### 3.4. Molecular Modeling and Simulation

Homology models of hTyr were constructed using the web-based I-Tasser server [27]. For further details, see text. Side-chain minimization and molecular dynamics (MD) simulations with hTyr models were carried out using the Amber force field as implemented in HyperChem 7.5 (Hypercube, Inc., Gainesville, FL, USA). Loops C2 → C3 and C4 → C5 (cf. Figure 3a) were subjected to 50 ps MD simulations at 300 K with heating- and cooling times of 5 ps each, step size 0.1 fs. The lowest-energy conformations observed during the runs were kept and energy minimized. Calculations of partition coefficients (logD) and protonation states were performed with Marvin (ChemAxon, Cambridge, MA, USA). Molecular docking simulations were performed using Molegro Virtual Docker (MVD, Molegro, Aarhus, Denmark). Protein targets and ligands for were prepared using the respective MVD subroutines. All amino acid side chains within 8 Å of the active site cavity were allowed to be flexible during the simulations; only the di-copper center was kept rigid. From the potential binding sites (“cavities”) proposed by MVD only those including the metal center were taken into account. For each compound, 20 runs with a maximum number of 500 iterations were carried out. The top ranking poses were kept and further refined using the Ligand Energy Inspector subroutine of MD. Discovery Studio Visualizer 4.0 (Accelrys, San Diego, CA, USA) was used for visual data analysis and presentation.

## 4. Conclusions

We show here that many thiazolyl resorcinols are very effective inhibitors of human tyrosinase in vitro. We further propose a structural model of the hTyr active site that is largely compatible with our inhibition data. Such information may greatly facilitate the design of novel inhibitors with even better efficacy in vivo. However, for a compound to qualify as ingredient of topical preparations for the treatment of skin hyperpigmentation, other properties are as important, including chemical stability, polarity, bioavailability, and half-life in vivo. For example, thiazolyl resorcinyl amides are generally more stable than amines in formulations. Furthermore, many compounds (especially those containing carboxyl groups) are highly effective against the isolated enzyme but not in melanocyte culture, because they do not penetrate the cell membrane. Clearly, more research is needed to explore the full potential of thiazolyl resorcinols for the treatment of human skin dyspigmentation.

## Figures and Tables

**Table 1:** Inhibitory activities (as EC_50_) of thiazolyl resorcinols inhibitors with modified rings. (**a**) Influence of modifications of the resorcinol ring; and (**b**) effects of replacement or modification of the aminothiazole moiety. The EC_50_ for kojic acid (W056) is shown for comparison.

(a)			(b)		
Code	Structure	EC_50_ (µM)	Code	Structure	EC_50_ (µM)
**W311**	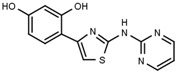	3.2 ± 0.1	**W547**	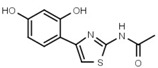	3.2 ± 0.6
**W452**	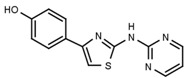	19 ± 1	**W633**	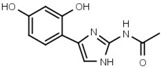	750 ± 60
**W539**	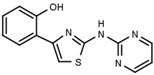	>3000	**W548**	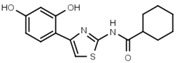	4.6 ± 0.2
**W568**	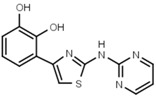	>3000	**W632**	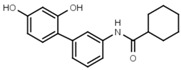	>3000
**W480**	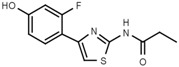	>3000	**W607**	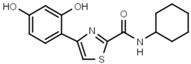	56 ± 3
**W624**	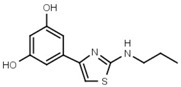	>3000	**W701**	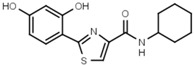	>3000
**W056**	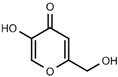	520 ± 50			

**Table 2:** Effect of the substituent at the 2-amino group of the thiazole moiety on measured and predicted inhibitory activity in (**a**) amines and amides; (**b**) Influence of size and hydrophobicity of terminal substituents on inhibitory activity. nrSc—negative rerank Score estimated by Molegro Virtual Docker (MVD).

Code	Core Structure					EC_50_ (µM)	nrSc
**W495**	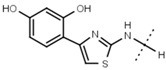					51 ± 2	86
**(a)**							
**Code**	**Amines**	**EC_50_ (µM)**	**nrSc**	**Code**	**Amides**	**EC_50_ (µM)**	**nrSc**
**W366**	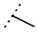	33 ± 1	-	**W547**	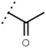	3.2 ± 0.6	102
**W538**	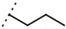	5.6 ± 1.4	-	**W577**	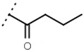	3.5 ± 0.5	-
**W533**	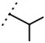	6.2 ± 0.2	-	**W630**	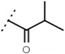	1.1 ± 0.1	114
**W534**	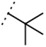	25 ± 1	-	**W687**	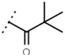	1.4 ± 0.1	-
**W688**	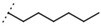	60 ± 2	-	**W695**	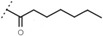	15 ± 1	-
**W694**	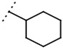	16 ± 1	93	**W548**	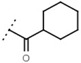	4.6 ± 0.2	-
**W367**	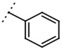	10 ± 1	-	**W498**	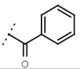	2.5 ± 0.1	-
**W693**	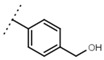	5.7 ± 0.2	-	**W619**	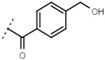	1.6 ± 0.4	-
**(b)**							
**Code**	**R**	**EC_50_ (µM)**	**nrSc**	**Code**	**R**	**EC_50_ (µM)**	**nrSc**
**W688**	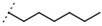	60 ± 2	-	**W692**	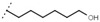	6.9 ± 0.2	-
**W669**	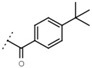	81 ± 5		**W680**	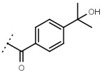	3.2 ± 0.2	-
**W619**	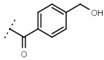	1.6 ± 0.4	-	**W696**	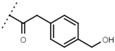	6.5 ± 0.2	-
**W542**	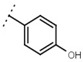	9.8 ± 0.3	105	**W693**	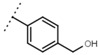	5.7 ± 0.2	-
**W367**	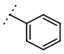	11 ± 1	-	**W496**	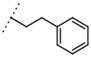	140 ± 6	86
				**W537**	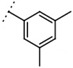	160 ± 2	-
**W529**	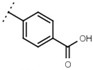	2.6 ± 0.1	-	**W570**	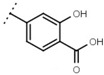	3.5 ± 0.1	115

**Table 3:** Inhibitory activities (as EC_50_) of (**a**) resorcinol and 4-alkyl resorcinols; and (**b**) methylated thiazolyl resorcinols. nrSc—negative rerank Score estimated by MVD.

(a)				(b)		
Code	Structure	EC_50_ (µM)	nrSc	Code	Structure	EC_50_ (µM)
**W089**	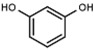	>3000	48	**W548**	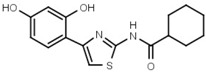	4.6 ± 0.2
**W039**	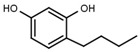	21 ± 4	92	**W605**	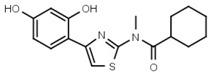	1400 ± 160
**W785**	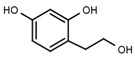	560 ± 90	66	**W625**	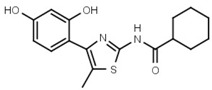	>3000
**W072**	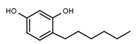	93 ± 21	88	**W681**	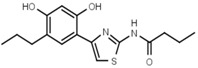	>3000
**W038**	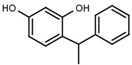	130 ± 10	84	**W694**	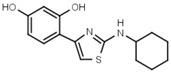	16 ± 1
**W639**	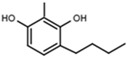	220 ± 10	-	**W646**	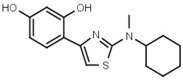	40 ± 2

## Supplementary Materials

Supplementary materials can be found at http://www.mdpi.com/1422-0067/19/3/690/s1.

## Abbreviations

bTyr	bacterial tyrosinase from *B. megaterium*
hTyr	human tyrosinase
mTyr	mushroom tyrosinase from *A. bisporus*
sTyr	bacterial tyrosinase from *S. castaneoglobisporus*
hTrp-1	human tyrosinase-related protein 1
TM-score	template modelling score
MVD	Molegro Virtual Docker
nrSc	negative rerank score as calculated by MVD
RSQ	residue-specific quality of an I-Tasser model
RMSD	root mean square deviation
