# Supplementary material for: Structure-Activity Relationships of Thiazolyl Resorcinols, Potent and Selective Inhibitors of Human Tyrosinase
Source: Int J Mol Sci. 2018 Feb 28;19(3):690. doi: 10.3390/ijms19030690 (PMC5877551; doi:10.3390/ijms19030690)
Supplement: Supplementary file 1 [file ijms-19-00690-s001.pdf]

Supplementary Materials

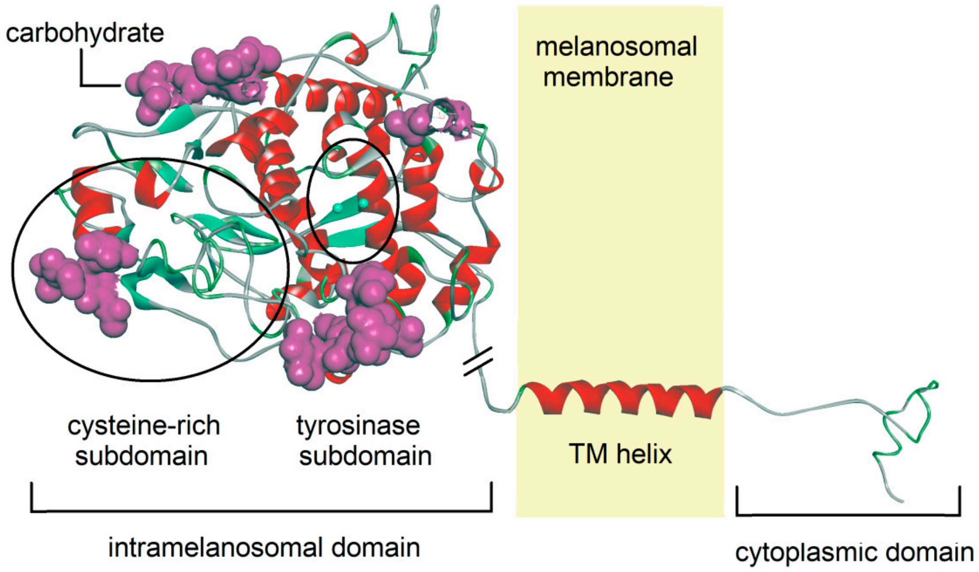

Figure S1. Architecture and subcellular location of human melanogenic proteins. The structure of the intramelanosomal domain corresponds to that of human tyrosinase-related protein 1 (hTrp-1, PDP entry 5M8L), while the remaining part of the molecule is depicted in a semi-schematic way. Carbohydrate chains present in the hTrp-1 structure are shown as CPK models in magenta. The metal ions residing in the active sites are depicted as cyan balls.

Table S1. Energetic contributions of partial structures to the total interaction energy as calculated by MVD. The values given for selected compounds correspond to the sum of the atom interaction energies for the partial structures shown (as MolDock scores and as % of total, respectively).

Code	Interaction Energy *	Interaction Energy *	Interaction Energy *	Sum	EC_{50} (μM)
**W630**	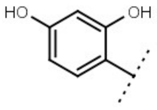 −63.5 (57%)	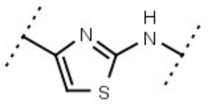 −36.0 (33%)	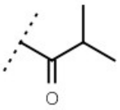 −10.9 (10%)	110	1.1
**W570**	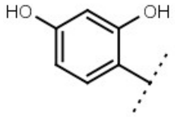 −66.5 (54%)	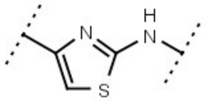 −36.7 (30%)	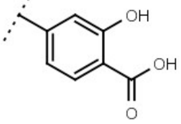 −19.0 (16%)	122	3.5
**W039**	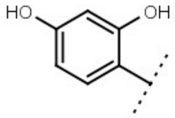 −61.9 (72%)	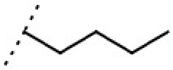 −24.5 (28%)	–	86	21
**W785**	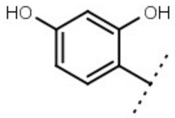 −67.7 (82%)	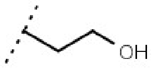 −14.8 (18%)	–	83	560
**W653**	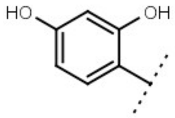 −40.0 (46%)	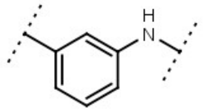 −33.7 (39%)	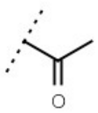 −12.7 (15%)	87	962
**W652**	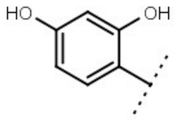 −52.4 (52%)	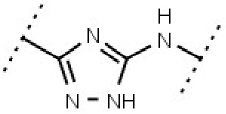 −37.7 (37.1%)	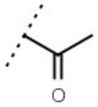 −11.5 (11.3%)	101	>3000

Synthesis Procedures of Alkylamidothiazoles Selected by Way of Example:

2-Bromo-2′,4′-bismethoxycarbonyloxyacetophenone:

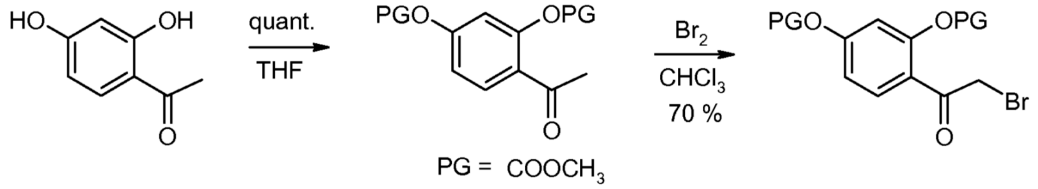

A solution of 60 g (369 mmol) of 2,4-dihydroxyacetophenone and 186 mL of triethylamine in 900 mL of tetrahydrofuran was cooled to 0 °C, and 93 mL of methyl chloroformate in 400 mL of tetrahydrofuran was slowly added dropwise. A white precipitate is formed. After stirring for 3 h at room temperature, the reaction is complete (TLC control). The precipitate was filtered off with suction and washed with copious amounts of tetrahydrofuran. The filtrate was evaporated to dryness on a rotary evaporator, taken up in ethyl acetate, washed with 1 N HCl and NaCl solution (sat.) and dried over magnesium sulfate, filtered from the magnesium sulfate, and the ethyl acetate was concentrated on a rotary evaporator. This gave 105 g of 2,4-bismethoxycarbonyloxyacetophenone. ^{1}H NMR (DMSO-d_6): 8.05 (d, 1H), 7.38 (d, 1H), 7.36 (s, 1H), 3.86 (d, 6H). The product was used without further purification. 63 g (392 mmol) of bromine in 450 mL of chloroform were added dropwise to the solution of 105 g of 2,4-bismethoxycarbonyloxyacetophenone in chloroform (1000 mL) over the course of 3 h. The reaction was then stirred for a further 15 min at room temperature. The solvent was evaporated on a rotary evaporator. The residue was stirred in ethyl acetate/n-hexane, and the resulting precipitate was filtered off with suction. Recrystallization from ethyl acetate/n-hexane produced 100 g of 2-bromo-2′,4′-bismethoxycarbonyloxyacetophenone. ^{1}H NMR (DMSO-d_6): 8.11 (d, 1H), 7.42 (m, 2H), 4.87 (s, 2H), 3.87 (s, 3H), 3.85 (s, 3H) ppm; m.p. 73-74 °C.

W630: N-(4-(2,4-Dihydroxyphenyl)thiazol-2-yl)isobutyramide:

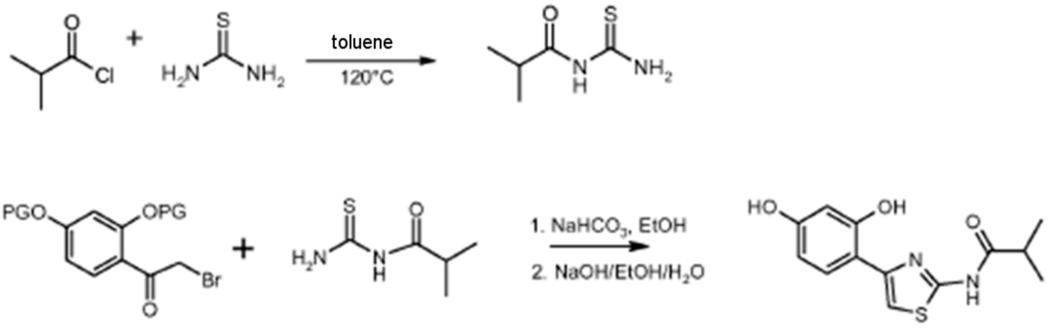

114 g (1.5 mol) of thiourea were introduced into toluene (800 mL), and 80 g (0.75 mol) of isobutyryl chloride were added dropwise. The reaction solution was boiled under reflux for 3 h, during which two phases formed. The upper phase was decanted off and cooled. The precipitated white crystals were filtered off with suction and washed with toluene and dried in vacuo. Yield: 62 g. ^{1}H NMR (DMSO-d_6): 11.03 (bs, 1H), 9.66 (bs, 1H), 9.35 (bs, 1H), 2.72 (m, 1H), 1.03 (2, 6H) ppm.

89 g (260 mmol) of 2-bromo-2′,4′-bismethoxycarbonyloxyacetophenone were boiled under reflux with 37.5 g (260 mmol) of *N*-isobutyrylthiourea and 32 g (380 mmol) of $NaHCO_3$ in 1000 mL of ethanol for 0.5 h. The reaction solution was cooled and admixed with 41 g (0.93 mol) of NaOH in 250 mL of water. After stirring for 30 min at room temperature, the reaction solution was taken up with 300 mL of water and adjusted to pH = 3 with 2 N HCl. The resulting precipitate was filtered off and recrystallized from ethanol/water. 56 g of thiazole were obtained. ^{1}H NMR (DMSO-d_6): 12.16 (bs, 1H),

10.88 (bs, 1H), 9.47 (bs, 1H), 7.65 (m, 1H), 7.41 (s, 1H), 6.32 (m, 2H), 2.75 (m, 1H), 1.14 (d, 6H) ppm; m.p. 243–245 °C.

W548: N-(4-(2,4-Dihydroxyphenyl)thiazol-2-yl)cyclohexanecarboxamide:

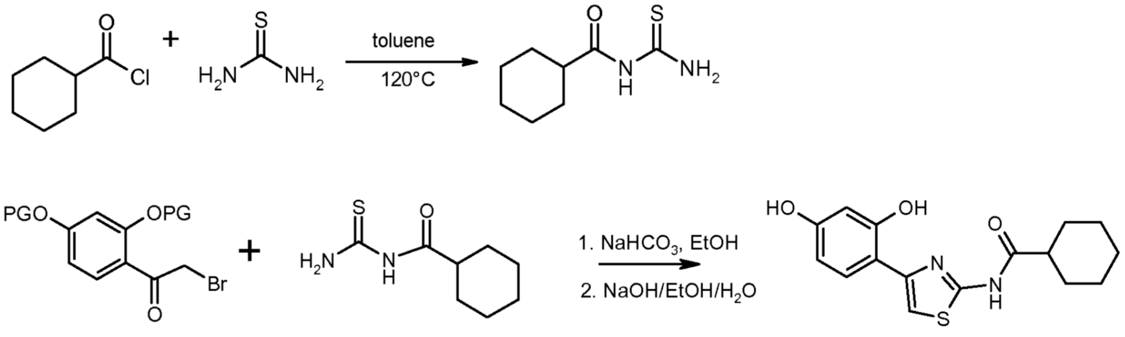

52 g (0.68 mol) of thiourea were introduced into toluene (500 mL), and 50 g (0.34 mol) of cyclohexanoyl chloride were added dropwise. The reaction solution was boiled under reflux for 3 h, during which two phases formed. The upper phase was decanted off and cooled. The precipitated crystals were filtered off with suction, washed with toluene and recrystallized from methanol. Yield: 35 g. ^{1}H NMR (DMSO-d_6): 10.98 (bs, 1H), 9.65 (bs, 1H), 9.32 (bs, 1H), 2.49 (t, 1H), 1.75 (m, 4H), 1.61 (m, 1H), 1.18 (m, 5H) ppm.

92 g (265 mmol) of 2-bromo-2′,4′-bismethoxycarbonyloxyacetophenone were boiled under reflux for 0.5 h with 49.4 g (265 mmol) of *N*-cyclohexanoylthiourea and 34 g (397 mmol) of $NaHCO_3$ in 900 mL of ethanol. The reaction solution was cooled and admixed with 37 g (930 mmol) of NaOH in 300 mL of water. After stirring for 30 min at room temperature, the reaction solution was taken up with 300 mL of water and neutralized with 2 N HCl. The ethanol was largely removed on a rotary evaporator. The precipitate formed was filtered off and recrystallized from ethanol/water. 70 g of thiazol were obtained. ^{1}H NMR (DMSO-d_6): 12.14 (bs, 1H), 11.00 (bs, 1H), 9.48 (bs, 1H), 7.64 (1 arom. H), 7.39 (s, 1H), 6.30 (2 arom. H), 2.49 (m, 1H), 1.84 (m, 2H), 1.76 (m, 2H), 1.65 (m, 1H), 1.42 (m, 2H), 1.25 (m, 3H), ppm; m.p.: 262–266 °C.

W696: N-(4-(2,4-Dihydroxyphenyl)thiazol-2-yl)-2-(4-(hydroxymethyl)phenyl)acetamide:

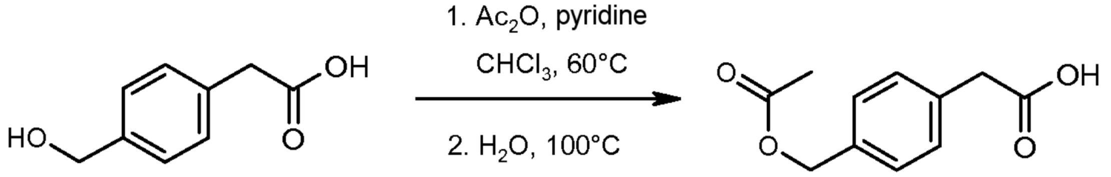

Procedure analogous to the literature: BANYU Pharmaceutical Co. Ltd., EP2072519 A1, 2009

Yield: 76%. ^{1}H NMR (DMSO-d_6): 12.31 (bs, 1H), 7.26 (m, 4H), 5.05 (s, 2H), 3.57 (s, 2H), 2.05 (s, 3H) ppm;

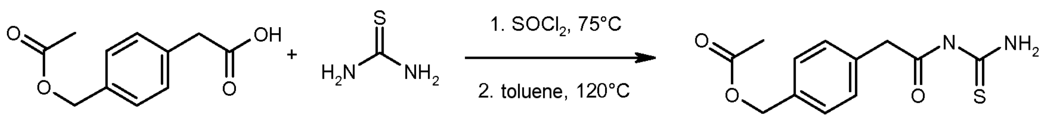

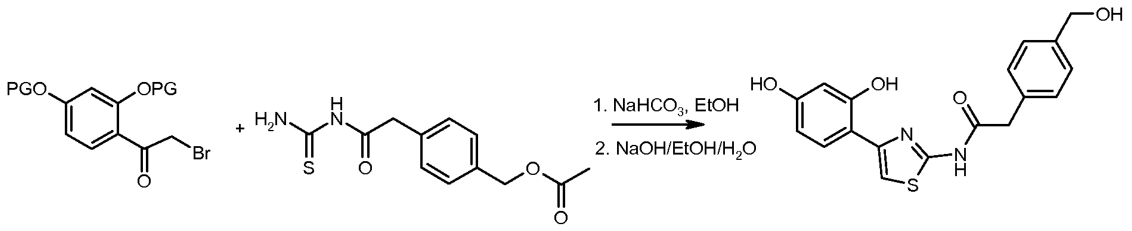

3.7 g (18 mmol) of 4-acetoxymethylphenylacetic acid were heated under reflux in 40 mL of thionyl chloride for 2 h. After removing the excess thionyl chloride in vacuo, the residue was taken up in 70 mL of toluene, and 2.7 g (36 mmol) of thiourea were added. The reaction solution was boiled under reflux for 3 h and then the solvent was removed in vacuo. Purification was by means of column chromatography with cyclohexane/ethyl acetate 1/1 on silica gel. Yield: 2.7 g. ^{1}H NMR (DMSO-d_6): 11.29 (bs, 1H), 9.55 (bs, 1H), 9.40 (bs, 1H), 7.30 (m, 4H), 5.04 (s, 2H), 3.71 (s, 2H), 2.05 (s, 3H) ppm;

3.5 g (10 mmol) of 2-bromo-2′,4′-bismethoxycarbonyloxyacetophenone were boiled under reflux for 0.5 h with 2.7 g (10 mmol) of *N*-[2-(4-acetoxymethylphenyl)acetyl]thiourea and 1.3 g (15 mmol) of $NaHCO_3$ in 50 mL of ethanol. The reaction solution was cooled and admixed with 4.0 g (0.1 mol) of NaOH in 20 mL of water. After stirring for 2 h at 60 °C, the reaction solution was taken up in 100 mL of water and adjusted to pH = 3 with 2 N HCl. The resulting precipitate was filtered off and recrystallized from ethanol/water. 1.3 g of thiazol were obtained. ^{1}H NMR (DMSO-d_6): 12.44 (s, 1H), 10.80 (s, 1H), 9.48 (s, 1H), 7.66 (d, 1H), 7.41 (s, 1H), 7.29 (m, 4H), 6.32 (m, 2H), 5.13 (t, 1H), 4.47 (d, 2H), 3.77 (s, 2H) ppm; m.p. 254–256 °C.
